# Ultrasound-Guided Erector Spinae Plane Block for Breakthrough Pancreatic and Hepatobiliary Malignancy Pain in the Emergency Department: A Case Series

**DOI:** 10.5811/cpcem.39723

**Published:** 2025-03-20

**Authors:** Richard J. Gawel, Jeffrey A. Kramer, Michael Shalaby

**Affiliations:** Hospital of the University of Pennsylvania, Department of Emergency Medicine, Philadelphia, Pennsylvania

**Keywords:** ultrasound-guided regional anesthesia, erector spinae plane block, cancer pain, pancreatic cancer, emergency department

## Abstract

**Introduction:**

Breakthrough pain is frequently experienced by patients with gastrointestinal malignancies and is a common reason for presenting to the emergency department (ED). After ruling out acute pathology, ED management typically consists of intravenous opioids, although high doses of opioids can be associated with potentially severe adverse events and complications in certain high-risk populations. Regional anesthesia strategies, such as the erector spinae plane block (ESPB), have been shown to be effective for several etiologies of non-malignant visceral abdominal pain. In this case series we sought to evaluate whether the ESPB can be effective for ED patients with breakthrough pancreatic and hepatobiliary cancer pain.

**Case Series:**

Three patients with breakthrough hepatopancreatobiliary cancer pain underwent successful ESPBs performed by an emergency physician in the ED. All patients reported considerable reduction in their pain. Two patients with cancer of the pancreatic head reported complete pain relief and were able to be discharged from the ED. The third patient with metastatic colorectal cancer involving the hepatobiliary system was admitted for further medical workup, although he did not require any additional analgesics for nearly 13 hours after the block.

**Conclusion:**

The erector spinae plane block appears to be a safe and effective strategy for managing breakthrough pain related to pancreatic and hepatobiliary malignancy in the ED.

## INTRODUCTION

Nearly half of all patients with gastrointestinal (GI) cancer suffer from chronic abdominal pain.^1^ In particular, pancreatic cancers and certain hepatobiliary malignancies are among the most painful cancers, with as many as 80% experiencing considerable pain.^2^ Even among patients with well-controlled background pain, more than half will have breakthrough pain. Cancer patients with breakthrough cancer pain frequently present to the emergency department (ED) for control of this pain. Breakthrough pain is the most common complaint for patients with cancer who present to the ED.^3^ Emergency department management typically consists of intravenous opioids, although many cancer patients still have considerable pain despite this treatment. Furthermore, opioids are associated with significant adverse effects, including dependence and tolerance, delirium, nausea and vomiting, and respiratory depression.^4^

The erector spinae plane block (ESPB) is a relatively new regional anesthesia technique that is increasingly being used for analgesia and anesthesia in the ED due to its favorable safety profile.^5^ Outside the perioperative setting, the ESPB is most commonly used for managing pain associated with rib fractures and has been effective in reducing pain and decreasing opioid utilization among hospitalized patients with rib fracture.^6^ A recent randomized controlled study of ED patients with acute hepatopancreatobiliary pain demonstrated that ESPBs performed by emergency physicians (EP) resulted in significantly lower pain scores and a reduced need for rescue analgesia than patients managed with standard analgesia alone.^7^ As has been documented in a few case reports, the ESPB has also been used to manage refractory breakthrough malignancy pain in patients with colon cancer^8^,^9^ and cholangiocarcinoma.^7^,^10^ We present a series of ED patients who underwent an EP-performed ESPB for refractory pancreatic and hepatobiliary malignancy pain.

## CASE SERIES

### Procedure

**Image 1:**
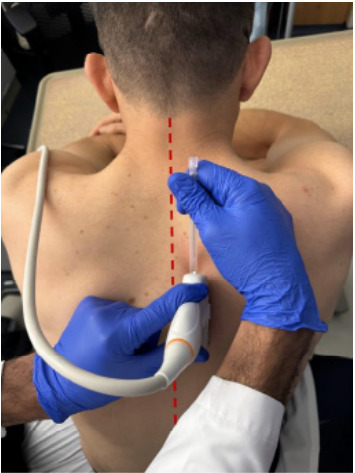
Patient set-up and probe orientation for thoracic erector spinae plane block. The patient is seated on the edge of the bed with arms resting on a side table. A curvilinear probe is oriented on the right paraspinal region with the indicator facing cranially, while the needle is inserted in a craniocaudal direction. Midline is indicated on the patient model with a dashed line.

The ESPB was performed as described in Forero et al 2016.^11^ After consenting for the procedure, patients were placed on a cardiac monitor and positioned seated on the edge of the bed. A 5–15 megahertz curvilinear abdominal probe was placed on the right posterior trunk just lateral to the midline in a caudal-cephalad orientation with the indicator facing cranially (Image 1). The erector spinae muscle and transverse processes were visualized with ultrasound, and the ultrasound probe was centered at the sixth thoracic vertebra. The surrounding area was prepped with chlorhexidine in standard fashion, and a small wheal of lidocaine was raised in the skin at the spinal needle insertion site. A Quincke spinal needle was introduced through the skin in a cephalad to caudad direction and advanced in-plane under sonographic visualization until the needle tip reached immediately deep to the erector spinae muscle, slightly superior to the transverse process. The syringe was aspirated to ensure the needle tip was not placed intravascularly.

**Image 2:**
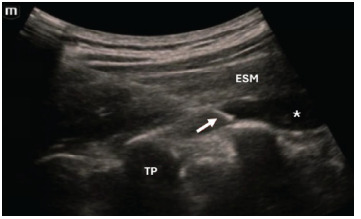
Sonographic anatomy of the erector spinae plane block. The arrow points to the needle tip, with the asterisk indicating spread of anesthetic injected within the fascial plane between the erector spinae muscle and the transverse process. The “m” in the upper left of the figure shows the location of the probe indicator, which is facing cranially relative to the patient. *ESM*, erector spinae muscle; *TP*, transverse process.

A small test injection of normal saline was used to confirm correct needle-tip placement within the fascial plane between the erector spinae muscle and the transverse process. Weight-based bupivacaine 0.5% was administered in 5 milliliter (mL) aliquots, while observing anesthetic spread within this fascial plane (Image 2). We expanded the volume of anesthetic by diluting it with normal saline to allow for maximal spread to target nerves, using between 15 and 30 mL of 0.9% normal saline. We opted for bupivacaine to allow for the greatest duration of analgesia. All patients were maintained on the cardiac monitor before blockade and were admitted to a telemetry unit.

### Cases

#### Case 1

A 67-year-old man with a known history of unspecified primary malignancy of the pancreatic head and chronic lymphocytic leukemia presented with acute on chronic epigastric abdominal pain, 10/10 in severity, refractory to his outpatient analgesic regimen. He was hemodynamically stable, and laboratory and imaging workup did not reveal any acute abnormalities. He underwent a right-sided ESPB with 20 mL of 0.5% bupivacaine mixed with 20 mL of normal saline without complications. Following the block, he reported complete (0/10) improvement in his pain. He was discharged home and did not return to the ED until approximately one month later when he had another episode of breakthrough cancer pain.

**CPC-EM Capsule**

What do we already know about this clinical entity?

*The erector spinae plane block (ESPB) is more frequently being used in the emergency department (ED) for thoracoabdominal wall and abdominal visceral pain*.

What makes this presentation of disease reportable?

*These are the first reported cases of an ESPB used for management of breakthrough pain from pancreatic cancer*.

What is the major learning point?

*The ESPB might be an effective analgesia strategy to manage breakthrough pain from pancreatic cancer and other abdominal gastrointestinal cancers in the ED*.

How might this improve emergency medicine practice?

*The ESPB may enable emergency physicians to manage breakthrough cancer pain, thereby reducing the amount of potentially harmful opioid analgesics*.

#### Case 2

A 68-year-old woman, also with a known history of unspecified cancer of the pancreatic head, presented with severe 10/10 acute on chronic epigastric pain attributed to her cancer. After an otherwise unremarkable ED workup, she underwent a right-sided ESPB with 15 mL of 0.5% bupivacaine mixed with 15 mL normal saline, which completely resolved her pain. She was discharged from the ED, although she returned approximately 48 hours later due to recurrence of her cancer pain.

#### Case 3

A 68-year-old man with a history of colorectal cancer and known metastases to the liver and biliary system presented with severe epigastric and right upper quadrant abdominal pain. He was initially treated with 2 milligrams of hydromorphone, which improved his pain from 10/10 to 8/10. Given this negligible improvement, he underwent a right-sided ESPB with 25 mL of 0.5% bupivacaine plus 25 mL of normal saline. Following the block, his pain improved to 3/10, although given the complexity of his cancer and concern for worsening involvement within the biliary system, he was admitted to the oncology service for pain control and additional medical workup. Notably, he did not require any additional analgesics until 13 hours after the block.

## DISCUSSION

In this case series, we demonstrate the successful use of the ESPB to manage breakthrough hepatopancreatobiliary cancer pain in the ED. To our knowledge, these are the first reported cases of EP-performed ESPBs used to manage breakthrough pain from pancreatic cancer. Moreover, we demonstrate another successful case of an EP-performed ESPB to manage refractory pain from colorectal carcinoma with hepatobiliary metastases.^8^ Following the ESPBs, both patients with breakthrough pancreatic cancer pain were able to be discharged from the ED, avoiding hospitalization for pain control. The latter patient with metastatic colorectal cancer was ultimately admitted for additional medical workup, although he did not require additional analgesics for 13 hours after the block. Given how frequently cancer patients seek emergency care for breakthrough pain, the ESPB may be a promising component of a multimodal analgesia strategy to manage breakthrough abdominal malignancy pain in the ED.

Compared to other truncal nerve blocks that only target the somatic innervation of the thoracoabdominal wall, the ESPB also provides sensory analgesia to visceral abdominal organs through blockade of the sympathetic chain.^12^ In an ESPB, local anesthetic is administered within the fascial plane between the erector spinae muscle and the transverse processes, where it can spread several vertebral levels cranially and caudally to anesthetize multiple spinal nerve roots with a single injection.^11^ Additionally, anesthetic simultaneously diffuses anteriorly into the paravertebral space to surround the sympathetic chain, thereby enabling blockade of sympathetic sensory afferents from the gastrointestinal tract.^13^ This allows the ESPB to provide analgesia for patients in significant pain from numerous abdominal visceral pathologies, including colorectal, hepatobiliary, pancreatic, and likely other sources of gastrointestinal malignancy.^7^–^9^ Since the afferent sympathetic neurons from the abdominal viscera converge prior to splitting into the left and right thoracic sympathetic chains, only a unilateral ESPB is required to provide analgesia.

Cancers of the pancreas and hepatobiliary system are notoriously painful and often poorly responsive to standard analgesia.^14^ As a result, a variety of multimodal analgesia strategies, including celiac plexus blocks, have been explored for managing refractory pancreatic cancer pain, although their efficacy has been somewhat inconsistent.^2^,^14^ These can occasionally lead to severe complications such as diaphragmatic paralysis, pneumothorax, retroperitoneal injuries, damage to abdominal viscera, and neurovascular damage due to the trajectory of the needle very close to several critical structures.^1^ The superficial needle trajectory in the ESPB and its reliable diffusion of anesthetic to the sympathetic chain may allow for safe visceral analgesia without the need for advanced imaging equipment or the expertise of interventional radiologists or endoscopists.

With the early success of the ESPB for managing visceral abdominal pain, it would be reasonable to consider augmenting these blocks with dexamethasone or other adjuvants to potentially prolong the duration of analgesia. For select patients who will ultimately require admission, EPs can also consider coordinating with anesthesiologists to place regional nerve block catheters in the ED to initiate continuous infusion of anesthetic. These strategies may allow EPs to initiate early, safe, and effective analgesia for cancer patients in the ED, which will continue to provide benefit well into their hospitalizations.

Caring for patients with GI malignancies with breakthrough cancer pain requires a multidisciplinary approach between the EP and the consulting oncologists and gastroenterologists. For patients awaiting admission to the hospital for continued medical care, the EP should coordinate with consultants prior to performing a block to inform them of the planned procedure and to elucidate any potentially unknown contraindications. This will not only improve the lines of communication between the ED and consultants to improve continuity of care but also ensure these patients receive appropriate monitoring in the hospital.

The ESPB should only be performed by EPs with either prior experience in regional anesthesia or at least experience with in-plane, ultrasound-guided needle placement, such as is used in placing peripheral intravenous lines. Although a rare complication, the ESPB can lead to pneumothorax, especially if an EP employs an overly acute needle trajectory that could allow the needle to enter the pleura. However, the naturally obtuse needle trajectory with the ESPB usually causes an overshot needle to back-wall to the next transverse process. Furthermore, all physicians who perform regional anesthesia, especially plane blocks such as the ESPB, which employ large volumes of anesthetic to allow for maximal spread to distant nerves, should always be aware of how to mitigate local anesthetic systemic toxicity. This can usually be avoided by calculating the local anesthetic dose based on the patient’s ideal body weight (maximum of 2.5 mg per kilogram for bupivacaine), aspirating prior to injecting, and only injecting increments of 5 mL of anesthetic at a time while monitoring for patient response. Lastly, patients being worked up for a surgical abdomen likely should not receive an ESPB due to masking their pain on physical exam, which may delay surgical treatment. However, patients who are being admitted for planned surgical intervention are still candidates for an ESPB for analgesia.

## CONCLUSION

The erector spinae plane block appears to be a safe and effective strategy for managing breakthrough pain related to pancreatic and hepatobiliary malignancy in the ED. Implementation of the ESPB in the ED for patients with visceral malignancy may improve pain, reduce the use of opioids, and in some cases avoid the need for hospitalization. Future studies should evaluate the efficacy of the ESPB on ED patients presenting with breakthrough pain from other abdominal visceral malignancies. Moreover, future investigation should aim to better compare the efficacy of the ESPB to conventional analgesia strategies among ED patients with breakthrough GI malignancy pain.
